# 多西他赛与吉非替尼序贯应用对人肺腺癌细胞SPC-A1生长及信号蛋白的影响

**DOI:** 10.3779/j.issn.1009-3419.2011.05.01

**Published:** 2011-05-20

**Authors:** 文颖 张, 为民 张, 林 王, 静娴 郑, 锋 肖

**Affiliations:** 1 510010 广州，广州军区广州总医院肿瘤科 Department of Medical Oncology, Guangzhou General Hospital of Guangzhou Military Command, Guangzhou 510010, China; 2 510182 广州，广州医学院研究生院 Postgraduate School, Guangzhou Medical Institution, Guangzhou 510182, China

**Keywords:** 肺肿瘤, 多西他赛, 吉非替尼, 细胞生长, 信号蛋白, Lung neoplasms, Docetaxel, Gefitinib, Proliferation, Signal transduction protein

## Abstract

**背景与目的:**

已经证明：化疗联合表皮生长因子受体酪氨酸激酶抑制剂（epidermal growth factor receptor-tyrosine kinase inhibitors, EGFR-TKIs）与单独化疗比较治疗晚期非小细胞肺癌并不能增加疗效，但机制尚未完全明了。本研究通过观察多西他赛与吉非替尼不同时序应用对人肺腺癌SPC-A1细胞生长及对EGFR及其信号蛋白ERK、AKT和胰岛素样生长因子（insulin-like growth factor, IGF）1型受体（IGF-1R）表达及磷酸化的影响，探索二者序贯用药增效的可能性及机制。

**方法:**

qPCR-HRM法检测细胞*EGFR*和*K-ras*基因突变；MTT检测细胞增殖；Western blot技术检测细胞EGFR、ERK、AKT、IGF-1R表达及磷酸化。

**结果:**

SPC-A1细胞*EGFR*和*K-ras*基因均无突变；与单药多西他赛或吉非替尼比较，多西他赛与吉非替尼同时应用及先吉非替尼后序贯多西他赛对细胞生长抑制作用均无明显差异，但先多西他赛后应用吉非替尼对其生长抑制作用明显增强。多西他赛和吉非替尼分别增强和抑制EGFR和ERK磷酸化。多西他赛诱导的EGFR和ERK磷酸化明显被序贯应用的吉非替尼抑制，但不能被同时应用的吉非替尼抑制。吉非替尼抑制EGFR和ERK磷酸化作用不能被同时或序贯应用的多西他赛逆转。多西他赛与吉非替尼不同时序应用对AKT及其磷酸化均无明显影响。多西他赛下调IGF-1R表达，吉非替尼对IGF-1R表达无明显影响。与多西他赛比较，多西他赛序贯吉非替尼对IGF-1R表达无明显影响。

**结论:**

多西他赛序贯吉非替尼对无*EGFR*突变的肺腺癌细胞SPC-A1生长抑制作用明显增强，其机制可能与影响细胞EGFR和ERK磷酸化有关，与AKT磷酸化及IGF-1R表达无关。

## Abstract (en)

**Background and objective:**

It has been proven that chemotherapy combined with epidermal growth factor receptor-tyrosine kinase inhibitors (EGFR-TKIs) could not increase response for advanced non-small cell lung cancer (NSCLC), but its cellular mechanism was not well known. The aim of this study is to assess the effects of sequential administration of docetaxel and gefitinib on the cell proliferation and signal pathway of lung adenocarcinoma cell SPC-A1 and its cellular mechanism.

**Methods:**

The mutation of *EGFR* and *K-ras* gene were examined by qPCR-HRM. MTT assay was used to measure the cell proliferation. The expression and phosphorylation of EGFR, ERK, AKT and IGF-1R were determined by Western blot.

**Results:**

No *EGFR* or *K-ras* gene mutation was found in SPC-A1 cells. Compared with docetaxel or gefitinib alone, no synergistic effects on the cell proliferation were observed in cells treated with docetaxel and gefitinib concomitantly or gefitinib followed by docetaxel. However, sequential administration of gefitinib following docetaxel could remarkably increase the inhibition of docetaxel on cell proliferation. Docetaxel increased, and gefitinib decreased, the phosphorylation of EGFR and ERK respectively. The suppression of pEGFR and pERK induced by gefitinib could not be activated by docetaxel, whether simultaneously or subsequently. No significant effects on the expression of AKT and p-AKT were found when docetaxel and gefitinib were administered simultaneously or sequentially. Docetaxel decreased the expression of IGF-1R.

**Conclusion:**

The phosphorylation of both EGFR and ERK, not the phosphorylation of AKT or the expression of IGFR, may contribute to the synergistic effects of EFGR-TKI following chemotherapy on the cell proliferation of NSCLC.

化疗和表皮生长因子酪氨酸激酶抑制剂（epidermal growth factor receptor-tyrosine kinase inhibitors, EGFR-TKIs）是晚期非小细胞肺癌（non-small cell lung cancer, NSCLC）的两大主要治疗手段，二者作用机制不同，理论上联合应用能产生增效作用，但多中心临床研究^[1, 2]^发现，化疗药物与EGFR-TKIs同时应用在疗效和生存上未优于单独化疗，先化疗后序贯应用EGFR-TKIs才能产生协同增效作用^[3, 4]^。同时应用未产生增效作用与EGFR-TKIs使细胞周期发生变化、降低细胞对化疗药物敏感性有关^[5, 6]^。但先化疗后序贯应用EGFR-TKIs产生协同增效作用与细胞周期变化无关^[7, 8]^，其确切细胞学机制有待阐明。

EGFR-TKIs通过抑制EGFR及其下游信号磷脂酰肌醇三羟基激酶/蛋白激酶B（phosphatidyl inositol -3-kinase and protein kinase B, PI3K/Akt）和丝裂原活化蛋白激酶/细胞外信号调节激酶1/2（mitogen-activated protein kinases/extra cellular signal-regulated kinases, MAPK/Erk1/2）传导，抑制肿瘤细胞增殖、侵袭、转移及凋亡。既往的研究^[8, 9]^显示，化疗药物与EGFR-TKIs均可影响EGFR及其下游信号传导蛋白表达，在因EGFR T790M突变对EGFR-TKIs产生继发耐药的NSCLC细胞中，化疗后序贯应用EGFR-TKIs产生协同增效作用与化疗药物影响EGFR磷酸化有关^[10]^，但对比例最高的EGFR野生型NSCLC细胞是否如此，目前未见报道。因此，本研究的第一个目的是应用EGFR野生型人肺腺癌SPC-A1细胞研究化疗药物与EGFR-TKIs不同序贯用药对细胞生长和EGFR及其下游信号传导蛋白表达变化关系，探讨二者序贯用药增效在EGFR野生型NSCLC细胞的可能细胞学机制。

研究^[11]^发现，包括NSCLC在内的多种恶性肿瘤均存在着胰岛素样生长因子（insulin-like growth factor, IGF）1型受体（IGF-1R）表达，IGF-1R和EGFR都通过激活PI3K/Akt和MAPK/Erk促进肿瘤细胞生长，在细胞信号传导和生理功能上存在相互作用、相互依存关系。IGF-1R表达与细胞对化疗及EGFR-TKIs敏感性相关^[11, 12]^，干扰细胞IGF-1R表达或IGF-1R抑制剂可以提高NSCLC细胞对化疗药物及EGFR-TKIs的敏感性，其增加化疗药物敏感性与抑制PI3K/AKT关系密切^[13, 14]^。反之，EGFR-TKIs影响IGF-1R表达^[12]^，化疗药物如多西他赛诱导肿瘤细胞调亡机制亦包括了对IGF-1R的抑制^[13, 15]^，化疗与EGFR-TKIs序贯治疗疗效是否与二者对IGF-1R蛋白信号通路影响有关，目前国内外未见报道。因此，本研究的第二个目的是检测多西他赛和吉非替尼不同给药时序对人肺腺癌细胞SPC-A1生长及细胞EGFR、ERK、AKT表达及磷酸化的影响，同时观测其对IGF-1R表达的影响，以探索IGF-1R表达在其细胞学机制中的作用，为目前疗效尚不理想的NSCLC探索新的治疗方向提供理论依据。

## 1 材料与方法

### 1.1 材料

人肺腺癌细胞SPC-A1由中科院上海细胞生物研究所提供。MTT、DMSO购自Sigma公司。吉非替尼和多西他赛分别由阿斯利康制药有限公司及江苏恒瑞公司提供，均溶于DMSO中，以5×10^-3^ mol/L储存于-20 ℃保存。IGF-1R一抗、β-actin、pEGFR一抗、ERK1/2一抗、pAKT一抗以及pERK1/2购自Cell Signaling公司，EGFR一抗、AKT一抗购自Santa Cruz公司，兔源及鼠源二抗购自Jackson Immuno公司，BCA试剂盒购自Pirce公司。ECL化学发光试剂盒购自GE Healthcare公司。DNA提取试剂盒购自天根生物公司。电泳仪及化学发光成像系统为Bio-Rad公司产品，酶联免疫检测仪为Biocell2010公司产品。

### 1.2 方法

#### 1.2.1 细胞培养

人肺腺癌细胞SPC-A1培养于含10%胎牛血清的RPMI-1640（Gibco公司）培养液，常规置于37 ℃、5%CO_2_培养箱中，细胞单层贴壁生长。实验取用生长状态良好的对数生长期细胞，胰酶消化传代，收集备用。

#### 1.2.2 细胞SPC-A1中*EGFR*和*K-ras*基因突变的检测

将处于对数生长期的细胞胰酶消化，离心重悬后按基因组DNA提取试剂盒说明操作提取DNA。取细胞SPC-A1 DNA产物8 μL进行电泳1.2%琼脂糖凝胶电泳检测提取效果。细胞*EGFR*及*K-ras*突变检测送由为真生物医药采用高分辨率溶解曲线分析技术（Quantitative PCR high-resolution melting, qPCR-HRM）完成。

#### 1.2.3 MTT法检测药物对细胞SPC-A1生长作用影响

取100 μL对数生长的SPC-A1细胞，按5×10^3^/孔接种至96孔培养板，每组4个平行复孔，细胞贴壁后，再按实验要求分别加入100 μL含有不同浓度的多西他赛和/或吉非替尼的培养基，按实验设计时间换含有不同药物的培养基，用酶标仪测定每孔细胞OD值。测定前向每孔内加入MTT 20 μL（5 g/L），孵育4 h后弃上清，加入150 μL DMSO，振荡15 min后酶标仪测定492 nm波长下各孔吸光值。计算药物作用细胞后存活率及其IC_50_值。实验分组如下：（1）对照组（无药物作用）（C）；（2）多西他赛单独作用72 h组（D）；（3）吉非替尼单独作用72 h组（G）；（4）同时给药组72 h（D+G）；（5）多西他赛作用24 h序贯吉非替尼48 h组（DG）；（6）吉非替尼作用48 h序贯多西他赛作用24 h（GD）。实验重复3次，生存率=（用药组OD值-空白对照OD值）/（对照组OD值-空白对照OD值）。

#### 1.2.4 Western blot检测药物对细胞信号蛋白的影响

细胞按设计分组给药处理后，将原培养基连同胰酶消化下的细胞离心，冷PBS洗3次，加入含有蛋白酶抑制剂和磷酸化蛋白酶抑制剂的裂解液冰上提取蛋白，BCA法测定蛋白浓度，等量上样，10%SDS-PAGE电泳，转至PVDF膜，5%牛血清白蛋白室温封闭2 h，一抗（1:400-1:1, 000）4 ℃摇床孵育过夜，TBST洗膜10 min×3次，二抗（1:3, 000）室温摇床孵育1 h，TBST洗膜10 min×3次，ECL显色后化学发光成像系统成像。以Band Scan图像分析软件进行积分光密度值分析。

### 1.3 统计学处理

采用SPSS 13.0软件进行统计学分析，各组间差异分析采用*One-way ANOVA*检验，以*P* < 0.05为差异具有统计学意义。

## 2 结果

### 2.1 SPC-A1细胞*EGFR*和*K-ras*基因状况

采用qPCR-HRM对人肺癌SPC-A1细胞的*EGFR*基因18、19、20、21外显子和*K-ras*基因2、3外显子进行突变检测，结果显示：样本均与阴性对照一致，无突变，SPC-A1细胞*EGFR*和*K-ras*基因均为野生型（图 1）。

**1:**
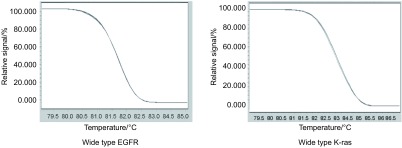
SPCA-1细胞*EGFR*和*K-ras*基因突变检测 Analysis of *EGFR* and *K-ras* gene mutation

### 2.2 多西他赛与吉非替尼对SPC-A1细胞生长影响

正常培养的肺腺癌SPC-A1细胞生长呈无限繁殖，第2天进入对数生长期（图 2）。多西他赛与吉非替尼具抑制SPC-A1细胞生长作用，作用呈时间依赖性和浓度依赖性。在一定浓度范围，多西他赛（1×10^-4^ mol/L-1×10^-12^ mol/L）与吉非替尼（1×10^-4^ mol/L-1×10^-10^ mol/L）浓度依赖性抑制SPC-A1细胞生长（图 3）。多西他赛和吉非替尼作用72 h对SPC-A1细胞抑制作用的IC_50_值分别为1.96×10^-7^ mol/L和2.01×10^-5^ mol/L。

**2:**
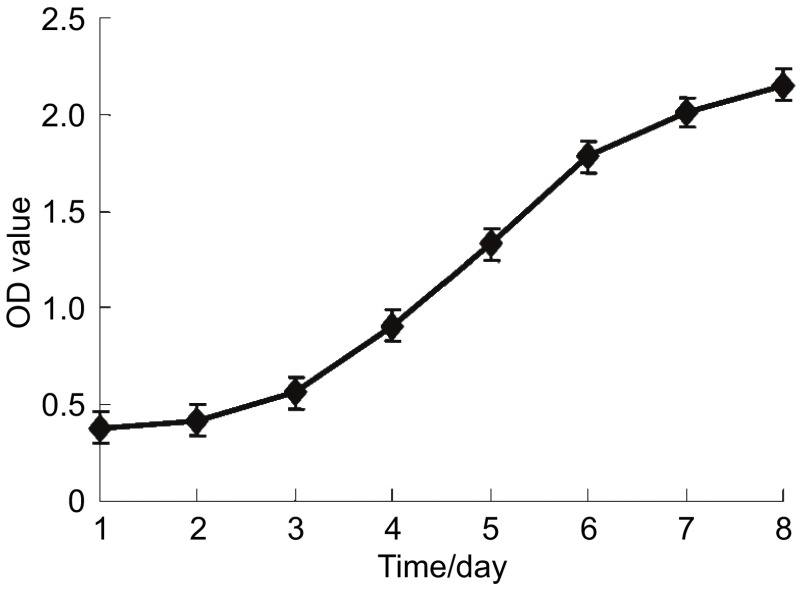
SPC-A1细胞的生长曲线 Proliferation curve of SPC-A1 cells

**3:**
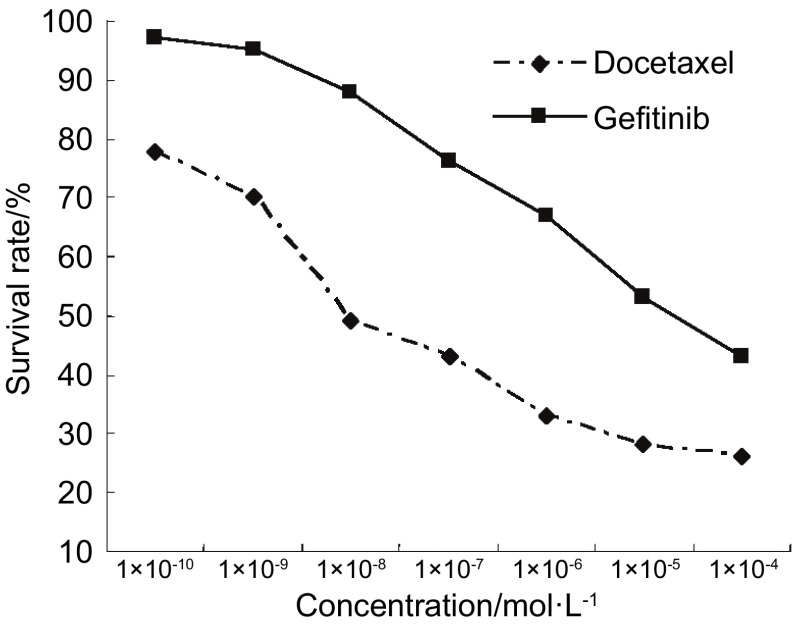
不同浓度吉非替尼和多西他赛对SPC-A1细胞生长的影响 Effects of gefitinib and docetaxel on the proliferation of SPC-A1 cells

### 2.3 多西他赛与吉非替尼同时及序贯应用对SPC-A1细胞生长影响

确定多西他赛和吉非替尼IC_50_值为两药同时及序贯应用浓度，作用72 h。多西他赛与吉非替尼对细胞生长SPC-A1存活率分别为（图 4）：多西他赛组（D）57.32%；吉非替尼组（G）54.94%；吉非替尼和多西他赛同时作用组（G+D）54.30%；多西他赛序贯吉非替尼组（DG）43.27%；吉非替尼序贯多西他赛组（GD）55.16%。吉非替尼和多西他赛同时作用组（G+D）及吉非替尼序贯多西他赛（GD）对细胞生长的抑制作用与单药组（G或D）无明显差别（*P* > 0.05）。多西他赛序贯吉非替尼组（DG）对细胞生长的抑制作用明显强于单独作用及同时给药组（*P* < 0.05）。

**4:**
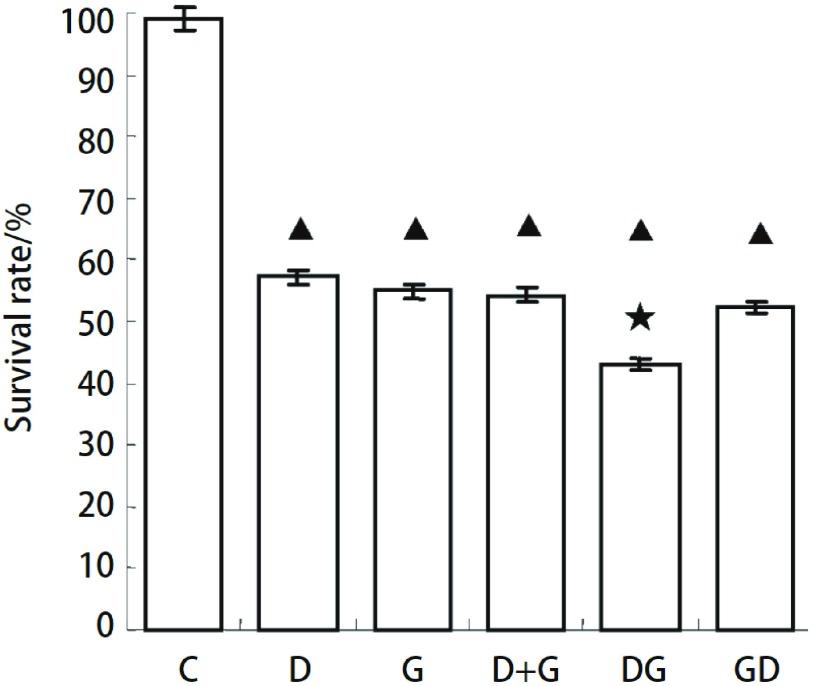
吉非替尼和多西他赛同时及序贯应用对SPC-A1细胞生长的影响。C：对照组；D：多西他赛组；G：吉非替尼组；D+G：多西他赛吉非替尼同时作用组；DG：多西他赛序贯吉非替尼组；GD：吉非替尼序贯多西他赛组。▲：与对照组相比，*P* < 0.05；★：与D和G组相比，*P* < 0.05 Effect of different combinations of gefitinib and docetaxel on the proliferation of SPC-A1 cells. C: Without treatment; D: Docetaxel alone; G: Gefitinib alone; D+G: Docetaxel plus Gefitinib; DG: Docetaxel followed by Gefitinib; GD: Gefitinib followed by Docetaxel. ▲: compare to control group, *P* < 0.05; ★: compare to D and G groups, *P* < 0.05

### 2.4 多西他赛与吉非替尼同时及序贯应用对SPC-A1细胞EGFR、ERK、AKT表达及其磷酸化、IGF-1R表达的影响

如图 5所示，多西他赛和吉非替尼对SPC-A1细胞非磷酸化的EGFR、ERK及AKT表达无明显影响；多西他赛和吉非替尼分别增强和抑制EGFR与ERK的磷酸化。相比多西他赛，多西他赛与吉非替尼同时应用EGFR和ERK磷酸化无明显变化，多西他赛诱导的EGFR和ERK磷酸化明显被序贯的吉非替尼抑制。相比吉非替尼，吉非替尼序贯多西他赛对EGFR和ERK磷酸化无明显变化。多西他赛与吉非替尼同时及序贯应用对AKT及其磷酸化无明显影响。多西他赛下调IGF-1R表达，吉非替尼对IGF-1R表达无明显影响。相比多西他赛单药，多西他赛序贯吉非替尼对IGF-1R表达无明显变化。

**5:**
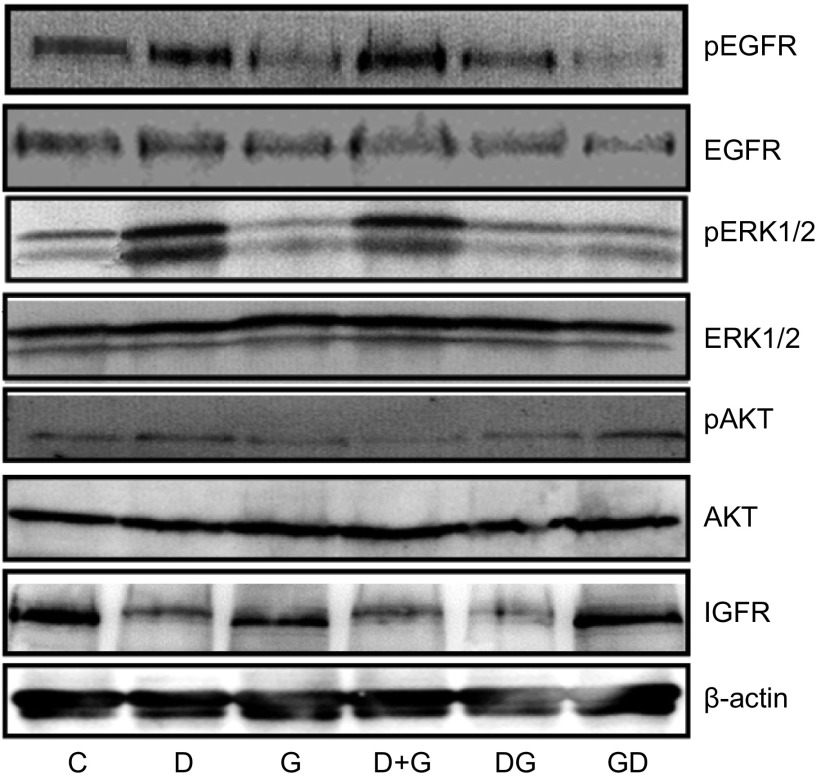
吉非替尼和多西他赛同时及序贯应用对SPC-A1细胞信号蛋白表达的影响 Effect of different combinations of gefitinib and docetaxel on the signal transduction protein expression of SPC-A1 cells

## 3 讨论

本研究结果显示，肺腺癌细胞株SPC-A1为EGFR野生型，多西他赛和吉非替尼在一定浓度范围均呈浓度依赖性抑制SPC-A1细胞增殖，SPC-A1为非EGFR-TKI敏感性细胞株，吉非替尼对其抑制作用较多西他赛弱，与文献^[16, 17]^报道一致。基于以下原由选用EGFR野生型细胞来进行研究：（1）既往发现化疗序贯EGFR-TKIs具协同增效作用的基础研究是来自EGFR野生型NSCLC细胞，临床研究来自*EGFR*突变状态未筛选的NSCLC患者；（2）EGFR-TKI对*EGFR*突变型细胞已具有很强的抑制能力，IPASS临床研究^[18]^显示吉非替尼对*EGFR*突变的NSCLC患者，有效率高达71%，化疗和EGFR-TKI合理联合应用对敏感性较差的EGFR野生型患者意义尤为重要。

本研究结果显示，先用多西他赛后序贯应用吉非替尼对细胞的生长抑制作用较多西他赛单药、吉非替尼单药、二者同时应用或先吉非替尼后序贯多西他赛明显，具协同增效作用，结果与Rosetti等^[19]^的报道一致。INTACT1、INTACT2、TRIBUTE和TALENT的多中心随机对照临床研究^[20, 21]^亦证实，对于*EGFR*突变状态未筛选的晚期NSCLC患者，化疗与EGFR-TKI同时应用未优于单独化疗。先化疗再序贯EGFR-TKI能够提高有效率，延长无疾病进展期及肺腺癌患者总生存^[4, 22]^。

已证实，EGFR-TKIs主要通过抑制PI3K-AKT和MARK/ERK这两个EGFR信号传导而抑制肿瘤细胞生长。本研究结果显示，吉非替尼抑制SPC-A1细胞生长的同时抑制EGFR、ERK的磷酸化水平，但未改变AKT的磷酸化，说明吉非替尼是通过抑制Raf/MEK/ERK这个信号通路抑制SPC-A1腺癌细胞增殖，而非通过抑制PI3K/AKT传导通路。对EGFR野生型的各种不同NSCLC细胞，吉非替尼对PI3K-AKT和Raf/MEK/ERK的抑制作用因细胞不同而异^[9, 23]^。

对化疗后序贯EGFR-TKIs协同增效作用的细胞学机制，既往的基础研究显示，在EGFR T790M突变对EGFRTKIs产生继发耐药的NSCLC细胞中，这种协同增效作用与化疗药物影响EGFR细胞内信号传导蛋白表达相关。本研究结果显示，多西他赛能明显诱导上调SPC-A1细胞EGFR、ERK磷酸化水平的结果与既往研究^[24, 25]^一致。除多西他赛外，其它化疗药物如紫杉醇、顺铂、吉西他滨和培美曲塞亦能上调NSCLC细胞EGFR及其信号蛋白磷酸化^[8, 9, 26]^。化疗药物诱导肿瘤细胞EGFR及其信号蛋白磷酸化也在白血病、前列腺癌、黑色素瘤和食道癌等许多肿瘤中得到广泛证实^[27, 28]^。化疗药物诱导肿瘤细胞信号蛋白磷酸化被认为是肿瘤细胞通过增强EGFR/ERK信号通路的活化进行增殖，逃避化疗的杀伤作用的重要方式^[28-30]^。本研究发现，先用多西他赛后序贯吉非替尼的EGFR和ERK磷酸化水平明显较多西他赛与吉非替尼同时应用或单独应用多西他赛低，这种EGFR和ERK磷酸化水平的变化不伴有EGFR和ERK的增加，结果说明化疗上调EGFR及ERK磷酸化，使吉非替尼抑制EGFR及ERK敏感性增强是化疗序贯吉非替尼的协同增效作用的细胞学机制之一。这一结果与Giovannetti^[8]^和Van Schaeybroeck等^[9]^的发现一致，化疗与EGFR-TKI联合应用于不同NSCLC细胞，只有细胞EGFR磷酸化（pEGFR）水平增加者才能从后续的吉非替尼治疗中获益，而与EGFR是否存在突变或扩增无关。本研究结果显示，相比多西他赛，吉非替尼与多西他赛同时应用或先吉非替尼后序贯多西他赛对细胞生长无明显影响，吉非替尼抑制EGFR和ERK磷酸化作用亦不能被同时或序贯应用的多西他赛逆转，结果说明多西他赛未增强吉非替尼抑制细胞生长作用可能与EGFR和ERK磷酸化水平低下有关。有研究^[31]^报道在结直肠癌细胞中下调EGFR磷酸化水平能导致EGFR-TKI与细胞毒化疗药物产生拮抗作用。

本研究SPC-A1细胞表达IGF-1R，与既往的报道^[32]^一致。化疗药物多西他赛能够明显抑制IGF-1R蛋白表达而吉非替尼对IGF-1R表达无影响，多西他赛与吉非替尼同时应用或先多西他赛后序贯吉非替尼均下调IGF-1R蛋白表达，结果初步说明多西他赛序贯吉非替尼的协同增效作用可能与IGF-1R表达无关，是否与IGF-1R及其细胞信号蛋白磷酸化有关我们正进行进一步实验研究。

本研究结果初步显示，化疗序贯EGFR-TKI对EGFR野生型NSCLC增效作用机制与化疗上调肿瘤细胞EGFR和ERK的磷酸化有关，与IGF-1R表达无关。值得一提的是，本研究只观察了一种EGFR野生型细胞，有待在多种NSCLC细胞中加以证实。
